# Sarcoid Nodular Myopathy Presenting With Hypercalcemia: A Rare Presentation—Case Report

**DOI:** 10.1002/ccr3.72675

**Published:** 2026-05-10

**Authors:** Vahideh Sadra, Amir Bahrami, Bahareh Mehramuz, Sina Hamzehzadeh, Mahsa Malekian

**Affiliations:** ^1^ Endocrine Research Center Tabriz University of Medical Sciences Tabriz Iran; ^2^ Clinical Research Development Unit, Sina Educational, Research and Treatment Center Tabriz University of Medical Sciences Tabriz Iran; ^3^ Student Research Committee Tabriz University of Medical Sciences Tabriz Iran

**Keywords:** hypercalcemia, sarcoid myositis, sarcoidosis, vitamin D

## Abstract

An 18‐year‐old female patient with no past medical history presented with nonanginal chest pain 2 months after her delivery. She did not report any other symptoms such as cough, dyspnea, nausea, or vomiting. Her vital signs were stable. The only positive finding on physical examination was tenderness and edema, with a palpable lump on her right thigh. Other examinations did not provide any additional information. Her lab results showed elevated calcium (12.5 mg/dL), low parathyroid hormone (5.6 pg/mL), and near‐normal 25‐(OH) Vitamin D levels (27.4 ng/dL). The core needle biopsy of the right thigh muscle revealed patchy lymphocytic infiltration with giant cells, without caseous necrosis, suggestive of sarcoidosis. The patient also had elevated angiotensin‐converting enzyme levels. The computed tomography of the lungs revealed no pulmonary involvement or hilar lymphadenopathy. The patient was treated with 30 mg of prednisolone daily. One month later, her symptoms improved, and calcium levels returned to normal.

## Key Clinical Messages

- Hypercalcemia can be the only manifestation of extrapulmonary sarcoidosis.
- Sarcoidosis should be considered in hypercalcemic patients with low parathyroid hormone levels.
- Musculoskeletal manifestations of sarcoidosis can occur in either acute myositis, chronic myopathy, or nodular myopathy.
- Sarcoidosis should be in the differential if the patient has hypercalcemia with suppressed PTH.

## 1 Introduction

Sarcoidosis is a noninfectious granulomatous disease characterized by the presence of noncaseating granulomas on biopsy [1]. Sarcoidosis has a spectrum of manifestations from acute to chronic or even asymptomatic presentation [2]. The most well‐known acute presentations are Löfgren syndrome and Heerfordt–Waldestrom syndrome [3]. Hypercalcemia is present in 7%–18% of cases [4]. Here, we represent a case of an 18‐year‐old female with extrapulmonary sarcoidosis initially presenting with hypercalcemia.

## 2 Case Presentation

An Azerbaijani 18‐year‐old female was admitted to the internal medicine ward with complaints of chest pain accompanied by pain and swelling in the lower extremities 2 months after delivery. She did not have a cough or dyspnea. The patient reported no other significant symptoms, such as weight loss or night sweats. There was no abnormality in the pregnancy period. There was no history of prior disease or medication use. She did not report a family history of disease. She described her chest pain as constant for over several hours, without any periods of relief. She did not report any increase in pain with exercise or radiation to any site. She had a heart rate of 115 beats per minute, with a blood pressure of 110/70 mmHg, and a respiratory rate of 19 per minute. On examination, the patient had a dry tongue and oral mucosa suggestive of volume depletion. Her heart and lung auscultation were normal, and no abnormality was observed on examination. She had tenderness and edema in the right thigh with a large lump on the anterior part of the same limb. No change in muscle tone or limb range of motion was observed. No abnormalities were found on neurological exams or abdominal examination.

## 3 Investigation and Treatment

Her laboratory test results revealed a white blood cell count of 9800/μL, with hemoglobin of 11.7 g/dL, and platelet count of 344,000/μL. She also had a creatinine of 0.8 mg/dL, TSH of 3.3 mIU/L (normal range: 0.5–5), erythrocyte sedimentation rate (ESR) of 24 mm/h, calcium level of 13.7 mg/dL (normal range: 8.5–10.5 mg/dL), albumin level of 4 g/dL (normal range: 3.5–5.5 g/dL), and creatine kinase of 250 U/L (normal range: up to 300 U/L). The patient's electrocardiogram was negative for ischemic changes, conduction blocks, or pericarditis. Cardiac troponin levels (hs‐CTnI) were requested for the patient and reported as < 14 ng/L. Echocardiography of the patient did not reveal any regional wall motion abnormalities.

The patient did not report any symptoms of hypercalcemia, such as constipation or a history of nephrolithiasis, and it was incidentally noted in laboratory testing. To evaluate the cause of hypercalcemia, we measured calcium levels simultaneously with parathyroid hormone (PTH), 25‐(OH) Vitamin D, and phosphorus. The results revealed a calcium of 12.5 mg/dL, with PTH levels of 5.6 (normal range: 12–65 pg/mL), 25‐(OH)‐vitamin D levels of 27.4 (deficiency < 20 ng/dL), and phosphorus levels of 2.9 (normal range: 2.5–4.5 mg/dL). Unfortunately, we couldn't measure the 1,25‐(OH)‐vitamin D levels in this patient since the equipment was unavailable. A malignancy workup for the patient was negative for multiple myeloma and any other malignancies.

**FIGURE 1:**
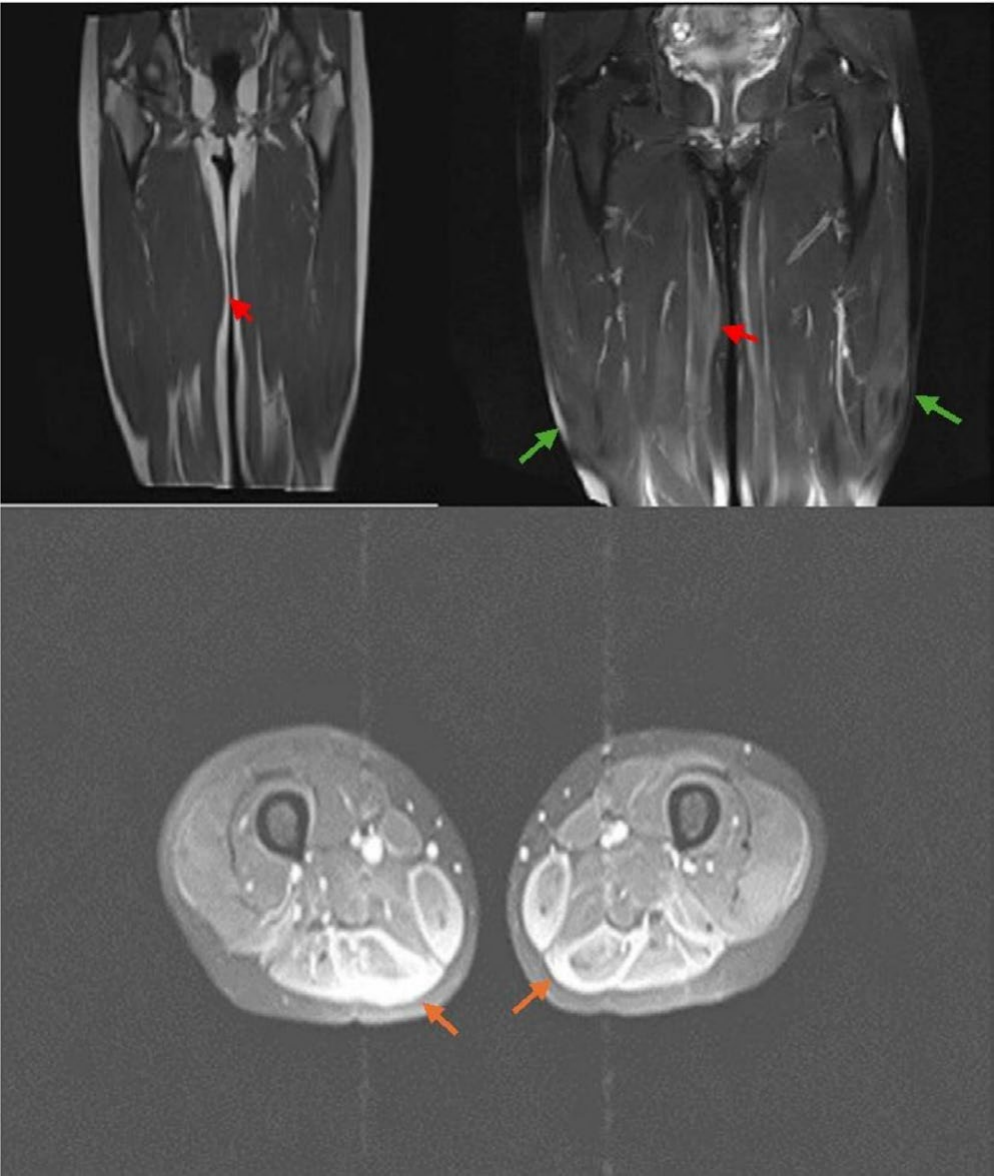
Soft tissue mass in the right limb (red arrow) and Mild soft tissue edema is seen at the bilateral vastus lateralis muscle (green arrow), with peripheral enhancement after contrast injection in the pes anserine muscle group (orange arrow).

Patient underwent a series of radiologic studies before biopsy to evaluate the origin of her mass in the right thigh. The ultrasound shows an 11 × 18 × 16 cm hypoechoic mass within the muscle. Magnetic resonance imaging (MRI) revealed mild bilateral soft‐tissue edema in the vastus lateralis muscles. Also, after contrast injection, bilateral peripheral enhancement was seen in the pes anserine muscle groups. Figure 1 shows the patient's MRI.

**FIGURE 2:**
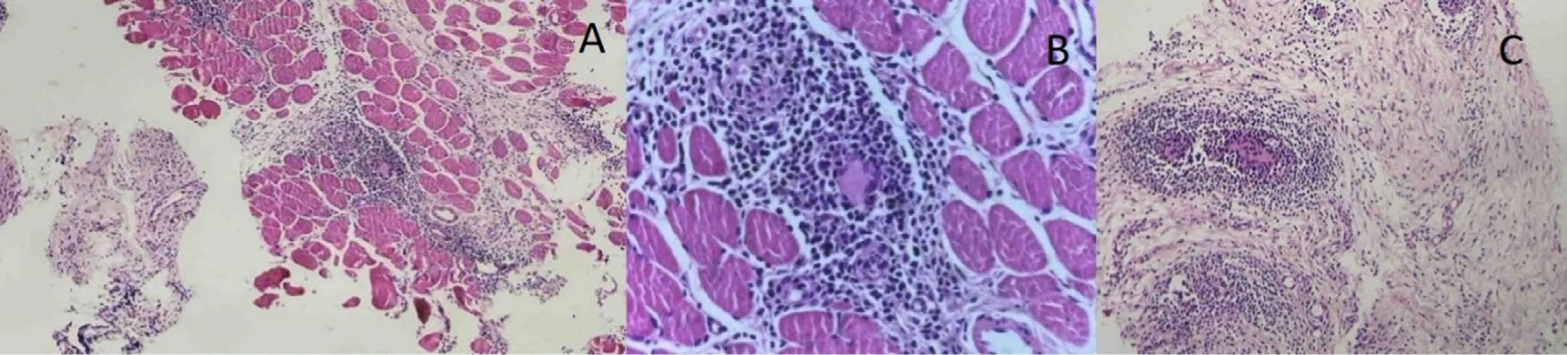
(A, B) Represent fibroconnective and muscle tissue with patchy infiltration of lymphocytes. (C) Represents the presence of giant cells.

The patient underwent an ultrasound‐guided core needle biopsy of the right thigh mass. The section shows fibroconnective and muscle tissue with patchy infiltration of lymphocytes and histiocytes associated with some giant cells in serial sections, without any caseous necrosis. The biopsy was compatible with sarcoidosis and is presented in Figure 2.

**FIGURE 3:**
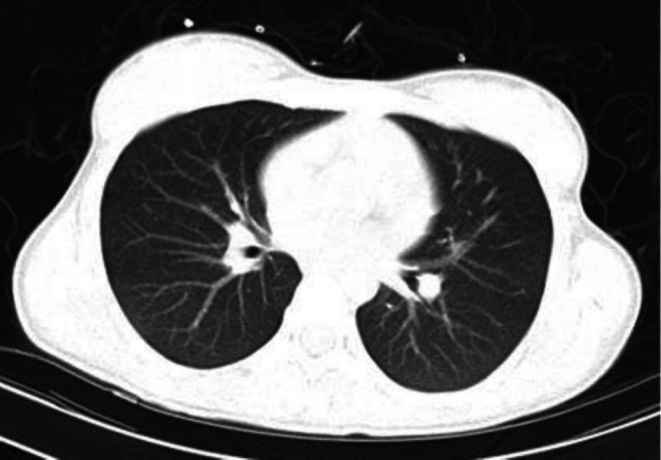
Computed tomography of the patient was negative for pulmonary sarcoidosis.

Angiotensin‐converting enzyme levels were measured at 119.5 IU/L (normal range: 13–63 IU/L), further strengthening the diagnosis. To evaluate for pulmonary sarcoidosis, a computed tomography (CT) scan was performed. No abnormality in the pulmonary parenchyma, no obvious interlobular septal thickness or nodules, and no lymphadenopathy in the hilar and mediastinal regions were noted. The patient's CT is shown in Figure 3.

The patient was treated at that time with IV fluid volume resuscitation and daily 30 mg prednisolone. Subsequently, her symptoms and serum calcium level were assessed to determine whether the treatment was effective.

## 4 Follow‐Up and Results

After 1 month, her symptoms improved, and her serum calcium level decreased to 9.2 mg/dL. Her limb was no longer painful. The prednisolone was continued for another 2 weeks with the same dose before reducing the dose to 10 mg over 8 weeks and maintained at that dose for another 3 months. No drug reaction and no complications were observed during treatment.

## 5 Discussion

Sarcoidosis is a multisystemic granulomatosis disease that most commonly affects the lungs; however, in 8.3% of patients, there is no pulmonary involvement [5]. Sarcoid myositis is a sporadic presentation, even among patients with extrapulmonary sarcoidosis. The differential diagnosis for sarcoid myositis includes inclusion body myositis, immune‐mediated necrotizing myositis, and steroid myopathy [6]. In a retrospective cohort study of 12 patients with sarcoid myositis, muscle edema followed by fat replacement was the most common MRI feature [7]. Tiger man sign for muscle sarcoidosis was present in 25% of cases [7]. The tiger man sign is mostly described in positron emission tomography (PET)‐based imaging techniques, due to stripes of hypermetabolic foci in muscle resembling a tiger‐man [8]. The “dark star” and the “three stripes” signs have been reported in MRI, but the sensitivity and specificity of them are not established [9]. In the muscle biopsy, both noncaseating granuloma and inflammation without granuloma were noted [7].

Approximately 50% of patients are reported to have granulomatous tissue in muscle without symptoms, and symptomatic muscle involvement is quite rare [10]. There are three main musculoskeletal presentations of sarcoidosis: acute myositis, chronic myopathy, and nodular myopathy [11]. Both acute myositis and chronic myopathy usually manifest with proximal muscle weakness. However, the chronic myopathy presents with a longer duration of weakness and may have normal creatine kinase levels in contrast to acute myositis [11]. Nodular myopathy, the rarest form among these three, appears as a soft tissue mass usually in the lower extremity [11]. In this subtype, creatine kinase and ESR levels may be normal and can be misinterpreted as those of a soft‐tissue tumor [9, 11, 12].

Hypercalcemia is a feature in 6%–18% of cases and is mainly attributed to high vitamin D levels [13]. The primary attributed mechanism is increased 1,25‐(OH)_2_‐vitamin D production by granulomatous tissue; however, some cases have been reported to occur due to PTH‐related peptide secretion [4, 13]. In both mechanisms, activated macrophages play the leading role in the pathogenesis of hypercalcemia [4]. Despite these facts, patients with sarcoidosis and hypercalcemia undertake a workup for other cases of hypercalcemia, such as malignancy. Low 25‐(OH)‐vitamin D was found to be associated with a chronic disease course, while high 1,25‐(OH)_2_‐vitamin D is associated with a chronic need for treatment [14, 15]. A limitation of our work is the lack of technical equipment to assess 1,25‐(OH)‐vitamin D levels. Corticosteroids, ketoconazole, hydroxychloroquine, and infliximab are all medical options in the treatment of sarcoidosis‐related hypercalcemia [16]. Proposed strategies to treat severe hypercalcemia in this population include rehydration with a 5 L saline infusion over the first 24 h (depending on fluid volume status), plus one dose of 4 mg zoledronic acid added to the solution, and 30 mg prednisolone [16].

## Author Contributions

**Vahideh Sadra:** conceptualization, investigation, writing – original draft. **Amir Bahrami:** conceptualization, investigation, writing – review and editing. **Bahareh Mehramuz:** investigation, visualization, writing – review and editing. **Sina Hamzehzadeh:** writing – original draft, writing – review and editing. **Mahsa Malekian:** investigation, project administration, writing – review and editing.

## Funding

The authors have nothing to report.

## Ethics Statement

This manuscript was approved by the Tabriz University of Medical Sciences ethical committee (IR.TBZMED.REC.1403.695). Written informed consent was obtained from the patient to publish the reports, the results of any evaluations, and related data.

## Consent

Written informed consent was obtained from the patient to publish any accompanying images.

## Conflicts of Interest

The authors declare no conflicts of interest.

## Data Availability

The datasets supporting the conclusions of this article are included within the article.
